# From Tumor Immunology to Immunotherapy in Gastric and Esophageal Cancer

**DOI:** 10.3390/ijms20010013

**Published:** 2018-12-20

**Authors:** David Vrána, Marcel Matzenauer, Čestmír Neoral, René Aujeský, Radek Vrba, Bohuslav Melichar, Nikol Rušarová, Marie Bartoušková, Janusz Jankowski

**Affiliations:** 1 Department of Oncology, Faculty of Medicine and Dentistry, Palacky University, Hnevotinska 976/3, 775 15 Olomouc, Czech Republic; davvrana@gmail.com (D.V.); mmatzenauer@me.com (M.M.); Bohuslav.Melichar@fnol.cz (B.M.); ni.ru@seznam.cz (N.R.); bartouskova.m@seznam.cz (M.B.); 2 Department of Surgery, Faculty of Medicine and Dentistry, Palacky University, Hnevotinska 976/3, 775 15 Olomouc, Czech Republic; Cestmir.Neoral@fnol.cz (Č.N.); rene.aujesky@gmail.com (R.A.); 3 National Institute for Health and Care Excellence, London SW1A 2BU, UK; prof.jankowski@outlook.com

**Keywords:** esophageal cancer, gastric cancer, immunotherapy, checkpoint inhibitors, microsatellite instability

## Abstract

Esophageal and gastric cancers represent tumors with poor prognosis. Unfortunately, radiotherapy, chemotherapy, and targeted therapy have made only limited progress in recent years in improving the generally disappointing outcome. Immunotherapy with checkpoint inhibitors is a novel treatment approach that quickly entered clinical practice in malignant melanoma and renal cell cancer, but the role in esophageal and gastric cancer is still poorly defined. The principal prognostic/predictive biomarkers for immunotherapy efficacy currently considered are PD-L1 expression along with defects in mismatch repair genes resulting in microsatellite instability (MSI-H) phenotype. The new molecular classification of gastric cancer also takes these factors into consideration. Available reports regarding PD-1, PD-L1, PD-L2 expression and MSI status in gastric and esophageal cancer are reviewed to summarize the clinical prognostic and predictive role together with potential clinical implications. The most important recently published clinical trials evaluating checkpoint inhibitor efficacy in these tumors are also summarized.

## 1. Introduction

Esophageal and gastric cancers are among the top ten most frequent and deadly tumors worldwide [1]. The etiology of both tumors is poorly understood, but bacterial, viral, and environmental factors are thought to play a substantial role. Since the percentage of adenocarcinoma of the esophagus is increasing rapidly over time (especially in Western countries where adenocarcinoma already is more common than squamous cell carcinoma) esophageal cancer will be discussed together with gastric cancer in which adenocarcinoma is the most prevalent histology. The treatment strategy, however, differs significantly. Primary surgical resection with adjuvant chemotherapy or chemoradiotherapy or perioperative chemotherapy are the principal treatment strategies for gastric cancer, while for esophageal cancer, neoadjuvant chemoradiotherapy followed by surgical resection is the current gold standard. Unfortunately, the prognosis of both disorders remains disappointing despite significant effort in the both clinical and preclinical research [2,3,4].

One of the fundamental evasion mechanisms of cancer cells, defined by Hanahan and Weinberg as “hallmarks of cancer”, is the alteration of the host immune system which allows tumor growth in a relatively hostile environment. The physiological role of the immune checkpoints is to prevent excessive immune response by termination of immune system activation at appropriate time. Tumor cells can use this mechanism to catalyze the auto-destruction of the immune response. These mechanisms are at the center of interest of the research aiming to sustain the activity of the immune system to allow the tumor cells to be destroyed, and acting on the immune checkpoints seems to be a promising option. Immunotherapy has established a firm position in the treatment of malignant melanoma, lung cancer, and clear-cell renal cancer, but its role in the treatment of esophageal and gastric cancer is much less defined [5,6,7].

The aim of the present review is to summarize the recent developments of immunotherapy in gastric and esophageal cancers with reference to key developments in other epithelial cancers.

## 2. Molecular Subtypes of Esophageal and Gastric Cancer

Esophageal squamous cancer is stratified into three distinct molecular subgroups based on the gene analysis. The Cancer Genome Atlas Research Network research group has evaluated 90 esophageal squamous cell carcinomas (ESCC). The first subgroup ESCC1 includes tumors, which respond poorly to chemoradiotherapy and are, generally, associated with poor prognosis. The principal genomic alteration identified is NRF2 pathway disruption. The second subgroup is ESCC2, characterized by the mutation of *NOTCH1, ZNF750, KDM6A, KDM2D, PTEN, PIK3R1*, and *CDK6* amplification. This subgroup is also associated with white blood cell infiltration. The last molecular subgroup ESCC3 is characterized by Phosphoinositide 3-kinase (PI3K) pathway disruption. Unfortunately, at present this stratification of squamous cell esophageal carcinoma lacks any specific clinical impact. At the same time, the proportion of MSI-H SCC is very low compared with gastric cancer [8].

Similarly, esophageal and gastroesophageal junction adenocarcinomas (EGADCs) have also been molecularly characterized. Secrier et al. [9] have reported the results of the whole-genome sequencing of 129 esophageal adenocarcinomas also distinguishing three major molecular subtypes. The first subgroup are the tumors with dominant C>A/T mutations, the second subgroup is mainly defective in homologous recombination/chromosome segregation, and the last subgroup has commonly T>G mutation with high mutation burden. Compared with the previous sub-classification of ESCCs, where clinical impact is minimal, the possible clinical importance in EGADC is obvious. The last subgroup of tumors with both high mutation loads and high number of neoantigens could be appropriate for the immunotherapy with checkpoint inhibitors. The second subgroup, similar to breast cancer patients with the *BRCA* mutation, could contain candidates for Poly (ADP-ribose) polymerase (PARP) inhibitors due to the disrupted homologous recombination. However, in the first subgroup of EGADC patients, chemotherapy remains the treatment of choice until further targeted therapy is available [9].

Last but not least, there is a gastric cancer sub-classification that was published in 2014 by the Cancer Genome Atlas Research Network. Gastric cancer was sub-classified into four major subtypes based on the molecular pattern. The most frequent subgroup of tumors is characterized by chromosomal instability (CIN) representing about 50% of all patients. This group of patients is characterized by TP53 mutation and RAS pathway disruption. Histologically this subgroup is characterized mostly by intestinal histology. Typically, there are several potential targets for the targeted therapy present (human epidermal growth factor receptor 2 (HER2), epidermal growth factor receptor (EGFR), tyrosine-protein kinase Met (MET)), but so far, except for trastuzumab, with limited clinical impact. The second most common subgroup are the tumors with microsatellite instability (MSI-H) representing about 22% of the patients. The evidence regarding the clinical impact of the MSI has been rapidly growing during the last few years and will be discussed in more detail below. The third subgroup of the tumors representing about 20% of the cases is called genomically stable (GC) tumors. Histologically, these tumors are characterized by diffuse histology and genetically by the *CDH1/RHOA* or *CLDN18-ARHGAP* mutations. The last subgroup of the tumors consists of tumors with Epstein-Barr virus (EBV) infection characterized by mutation of *PIK3CA*, *CDKN2A* silencing and *PD-L1/2* over-expression which is also discussed in more detail below [10].

## 3. Immune Checkpoints PD-1/PD-L1/PD-L2 and Clinical Significance in Gastric and Esophageal Cancer

Immune checkpoint receptor PD-1 is a co-inhibitory molecule that is physiologically expressed on immune cells such as T and B lymphocytes or myeloid cells providing the signal for the termination of immune system activity. PD-1 receptor has two natural ligands, PD-L1 (B7-H1) and PD-L2 (B7-DC), which can be expressed, in addition to immune cells, also in tumor cells, and represent a potential mechanism of immune surveillance escape [11].

Generally, *PD-L1* expression was considered to be a poor prognostic factor for patient outcome across different tumor types. However, it has been recently demonstrated that the situation is more complex, and PD-L1 may indicate protective as well as poor prognostic risks depending on the tumor subtype, disease stage, or prior treatment. Unfortunately, the predictive role of PD-L1 expression for the efficacy of immunotherapy did not meet clinicians’ expectations and is, in many settings, poorly defined.

The reports on the prognostic role of the PD-L1 expression in esophageal or gastric cancer are summarized in Table 1. The published data are highly heterogeneous in terms of disease stage (stage ranging from I to IV), treatment (surgery alone, neoadjuvant treatment, adjuvant treatment, or advanced line treatment), different methods of PD-L1 status evaluation (immunohistochemistry vs. RT-PCR), different antibodies which may recognize different parts of the PD-L1 and heterogeneity of the cells on which the expression was assessed (tumor cells, stromal cells, or lymphocytes), different cutoffs for positivity that ranges widely (1–50%) One of the potential pitfalls may also be associated with the fact that most of the trials were performed in Asian populations alone. The findings also reflect the heterogeneity of the patients enrolled in the trials ranging from better prognosis for patients expressing PD-L1 to worse prognosis. From the research papers reported there are, however, several issues that deserve special attention and have potentially clinical implications.

### 3.1. PD-L1 Expression and Radiotherapy

**Figure 1:**
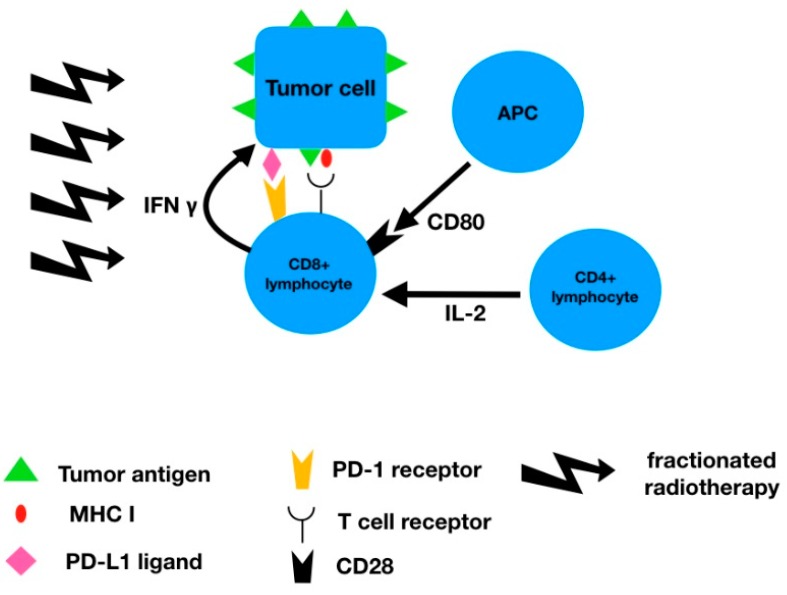
Connection of radiotherapy and PD-L1 expression.

Interesting findings were reported regarding radiotherapy and PD-L1 expression. Radiotherapy of localized esophageal cancer in neoadjuvant setting represents the gold standard of treatment. At the same time, adjuvant radiochemotherapy is the standard treatment for locally advanced gastric cancer after surgical resection. Zhang et al. have irradiated the esophageal squamous cell lines by using 6 MV linear accelerator with several 2 Gy fractions evaluating the PD-L1 expressing during the treatment. The data demonstrated an increasing expression of the PD-L1 during the treatment [19]. One of potential explanations of this observation is the induction of the immune response by the tumor destruction by the radiotherapy-induced immune response, and selection of PD-L1 expressing tumor cells. These findings are crucial in clinical practice since 2 Gy fractionation is, as mentioned above, the standard neoadjuvant/radical treatment approach. Considering these findings, immunotherapy could potentially improve outcome, specifically in the adjuvant setting. Several trials are currently investigating the role of immunotherapy in adjuvant setting after neoadjuvant chemoradiotherapy of esophageal/gastroesophageal junction cancer (CheckMate 577) [38] with no results reported so far, but in other tumor types, e.g., lung cancer, the administration of adjuvant immunotherapy (durvalumab) has resulted in significant improvement in patient outcome [39]. Additionally, PD-L1 expression seems to correlate with the response to neoadjuvant chemoradiotherapy and may be used to select patients who could potentially be spared from surgery after neoadjuvant chemoradiotherapy [40]. Finally, it has been demonstrated that the PD-L1 expression increases with increase in the total radiation dose. Years before programmed death receptor/ligands discovery, it was observed in the Intergroup 123 trial that an increased radiation dose (from 50 to 66 Gy) did not improve patient outcome in esophageal cancer [41], which was surprising considering the fact that radical doses for squamous cell cancer of other locations is usually close to 70 Gy. These findings were explained by the increased toxicity of the radiotherapy, but, considering the above-mentioned findings, one potential explanation could be an increasing resistance of the tumor cells associated with increased PD-L1 expression. Chen et al. have also reported significantly increased level of PD-L1 expression in patients not responding to neoadjuvant chemoradiotherapy compared with responders (72% versus 35%). This finding correlated with pathologic complete response achieved (16% versus 31%) [23]. The exact mechanism of increased PD-L1 expression is unknown, but there are several hypotheses. One of the viable explanations may be the increased secretion of interferon-gamma by the CD8+ T cell lymphocytes which stimulates PD-L1 expression as demonstrated by Dovedi et al. [42] (Figure 1). Interferon-gamma increases the PD-L1 expression through the Janus kinases-Signal Transducer and Activator of Transcription proteins (JAK-STAT) pathway. At the same time, increased PD-L1 expression is associated with epithelial-mesenchymal transition phenotype [43] which can further increase the tumor malignant (metastatic) potential.

These observations again highlight the fact that PD-L1 expression level is highly dependent on the previous treatment and makes its evaluation even more complicated.

### 3.2. PD-L1 Expression and Systemic Treatment

Another interesting point mentioned in these reports is the association of PD-L1 expression with the systemic treatment. The most frequently used chemotherapy regimens for both esophageal and gastric cancer include cisplatin, oxaliplatin, doxorubicin, 5-fluorouracil, paclitaxel, and docetaxel.

In contrast to radiotherapy in which PD-L1 expressions is increasing during the treatment and correlates with the total dose delivered, cisplatin-based chemotherapy in gastric cancer seems to decrease the expression of the PD-L1, specifically in responding tumors [26]. The simplest explanation for this could be the decreased total tumor mass, which corresponds with decreased PD-L1 positive tumor cells. However, the situation may be more complicated. Ghebeh et al. have shown that doxorubicin may translocate PD-L1 on breast cancer cells from the cell surface to the nucleus in association with the activity of the PI3K/AKT pathway [44]. Doxorubicin has been known to have immune-modulatory potential for decades and this can be potential explanation for this effect. Similarly, another anthracyclin epirubicin that is also used in gastric and esophageal cancer decreases PD-L1 level in breast cancer cell lines. On the other hand, some reports are suggesting that other cytotoxic agents such as paclitaxel can increase the expression of PD-L1 [45]. At the same time, 5-FU, probably the most widely used chemotherapeutic agent in gastric and esophageal cancer, was demonstrated to increase the PD-L1 expression as well [46]. Considering these observations, using immunotherapy may be more appropriate with or after taxane/5-FU-based chemotherapy. Again, chemotherapy regiments that have been used for a long time such as cisplatin/5-FU, carboplatin/paclitaxel, epirubicin/cisplatin/5-FU (ECF), or docetaxel/cisplatin/5-FU (DCF) could be also effective because of compensating effect on PD-L1 expression.

### 3.3. PD-L1 Expression and EBV Infection

Another frequently reported observation is the correlation of PD-L1 expression with EBV. EBV is human double stranded DNA virus (herpesviridae) which is a well-known cause of nasopharyngeal carcinoma or Burkitt lymphoma, but the relation with gastric cancer is less documented. Gastric cancer associated with EBV infections seems to more frequently express the PD-L1 and represent a disease with better prognosis. The mechanism for PD-L1 expression is probable related to latent membrane protein 1 (LMP1) and interferon-gamma stimulated by EBV which up-regulate the expression of PD-L1 [47]. EBV infection could potentially serve as another predictive marker for anti-PD-L1 directed immunotherapy not only in gastric cancer.

### 3.4. PD-L1 Expression and MSI Status

Importantly, MSI-H as a result of DNA mismatch repair gene deficiency is related to increased PD-L1 expression and better prognosis. The mechanism of PD-L1 over-expression in MSI-H situation can be explained by the fact that an increased number of neoantigens resulting from MSI-H phenotype attract a high number of T lymphocytes which stimulate PD-L1 expression through secretion of interferon-gamma [48].

### 3.5. PD-L1 Expression and Epithelial-Mesenchymal Transition Phenotype

Epithelial-mesenchymal transition (EMT) phenotype is an important step in the metastatic cascade of malignant tumors. EMT corresponds to the loss of epithelial cell phenotype (cell adhesion and polarity) and gaining of mesenchymal phenotype with aggressive potential. Gaining this potential seems to correlate with PD-L1 expression due to the presence in the promotor region of the PD-L1 of a binding site for Zinc finger E-box-binding homeobox 1 (ZEB1) transcription factor which is at the same time related to EMT. As a result of this dual action of ZEB1 protein, the tumors with PD-L1 expression and EMT phenotype have worse prognosis [49].

### 3.6. PD-L1 Expression and EGFR

The relationship between EGFR and PD-L1 expression is more complicated. EGFR expression seems to be immunosuppressive and correlates with low PD-L1 expression (again potentially explained by low interferon-gamma level due to lack of tumor infiltrating lymphocytes). The data showed that radiotherapy could induce PD-L1 expression and the administration of EGFR and EGFR inhibitors could counteract this aberrant PD-L1 expression [50]. Predictive role of PD-L1 is further discussed below.

PD-L2 has the same receptor as PD-L1, but its prognostic or predictive role is much less defined. The same applies for prognostic/predictive role of PD-1 receptor. From the published reports (summarized in Table 2) no prognostic value is apparent regarding the patient outcome as well as immunotherapy efficacy. 

## 4. Significance of Microsatellite Instability

Mismatch repair genes are the genes which replace nucleotides incorrectly incorporated during DNA replication. Damage of these repair genes was investigated in several tumor types and is also responsible for the most frequent hereditary form of colorectal cancer, i.e., Lynch syndrome. As a result of the defects in these genes (mismatch repair deficient phenotype; dMMR) there is a higher level of short repeated sequences of the DNA called microsatellites. High levels of microsatellite instability (MSI-H) are associated with several unique characteristics in relation to immunotherapy. Defects in DNA replication causes expression of defective proteins, which the immune system recognizes as neoantigens, and represents a potential target for immune cells. In the case of gastric cancer, the MSI-H phenotype correlates with high mutation burden (HMB) [53]. At the same time, attraction of the immune cells into the tumor environment is causing a high level of stimulatory cytokines (e.g., interferon-gamma). Taken all together, this constellation is pushing the immune balance towards immune stimulation and favorable clinical course. The principal immune cell populations responsible for tumor destruction are the CD3+ tumor infiltrating lymphocytes (TIL) including helper CD4+ and cytotoxic CD8+ lymphocytes while the principal suppressor cell subset is the FOXP3+ T cells [54]. It has been demonstrated in patients with colorectal cancer that the number of CD3+ and CD8+ immune cells is significantly increased in the MSI-H tumors, in contrast to FOX3+ T lymphocytes (T regs), whose level remains the same regardless of microsatellite instability [55].

In Table 3, a summary of reports evaluating the prognostic role of MSI-H in gastric cancer patients in presented. The percentage of MSI-H tumors in gastric cancer patients ranges from 8–25%, but in the case of squamous cell esophageal cancer the MSI-H phenotype is quite rare [56,57,58]. Generally, in patients with gastric adenocarcinoma MSI-H phenotype represents a positive prognostic factor regardless the disease stage. Some interesting results have been reported regarding adjuvant/perioperative chemotherapy and microsatellite instability. A retrospective analysis of the MAGIC trial evaluating perioperative ECF chemotherapy in gastric cancer reported no clinical benefit of perioperative chemotherapy in patients with MSI-H tumors [59]. It may be hypothesized that immunostimulatory environment of the MSI-H tumors itself can represent positive prognostic factor for patient outcome after surgery that cannot be further improved by the administration of systemic chemotherapy. Moving step further, it may be reasonable to further boost the already activated immune system by the administration of immune checkpoint inhibitors which could potentially neutralize the increase of PD-L1 expression by the tumor cells in response to immune stimulation. This theoretical assumption was confirmed clinical trials (Keynote-016, -164, -012, -028, and -158) that included patients with MSI-H tumors (mainly colorectal cancer, but also including gastric cancer patients). The objective response rate was 39.6%, which brought the US Food and Drug Administration (FDA) approval for pembrolizumab in several solid tumors including gastric cancer [60]. However, so far, there are no other trials confirming these results, and it should be also taken into consideration that the approval is based on just a few gastric cancer patients.

## 5. Immunotherapy

There are several current clinical trials, mostly with checkpoint inhibitors, investigating the role of immunotherapy in gastric and esophageal cancer. As mentioned above, there are subtypes of gastric cancer that could potentially be candidates for immunotherapy, including MSI-H tumors harboring multiple neoantigens resulting from mismatch repair gene deficiency as well as EBV-positive tumors expressing PD-L1/L2. These potentially immunogenic tumors represent about one-third of all tumors. The role of MSI phenotype was investigated in a phase I clinical trial where 15 tumor types were treated by pembrolizumab with promising results. The FDA has approved pembrolizumab based on this clinical trial for gastric cancer tumors with MSI-H phenotype [35]. However, we must be cautious about an over-interpretation of the results because only 5 patients with gastric cancer were included in this trial and most of the patients assessed had colorectal cancer. Thus, further trials are needed to confirm the role of immunotherapy in MSI-H gastric cancer. As already mentioned above, the incidence of MSI-H phenotype in esophageal cancer is very low so the investigation of immunotherapy should be supported by a different rationale, e.g., squamous histology. Another potentially predictive factor is the PD-L1 expression. Keynote-012 was a phase Ib trial enrolling patients with PD-L1 positive gastric and gastroesophageal (GE) junction cancer who were treated with pembrolizumab 10 mg/kg biweekly until 24 months, progression, or unacceptable toxicity. In total, 39 patients were enrolled in this trial that showed promising 22% overall response rate and triggered the initiation of further trials [70]. The phase II trial Keynote-055 that followed had three cohorts of patients with recurrent or metastatic gastric or GE junction adenocarcinoma, including pretreated patients treated by pembrolizumab 200 mg triweekly; patients treated in the first line with cisplatin and 5-FU or capecitabine in combination with pembrolizumab 200 mg triweekly and patients treated in the first line with pembrolizumab 200 mg triweekly alone. The trial showed overall response rate of 11.6% with median duration of the response 8.4 months. However, when patients were evaluated according to PD-L1 expression, overall response rate (ORR) was 15.5% in PD-L1 expressing tumors and 6.4% in PD-L1 negative tumors with median duration of response of 16.3 months in in patients with PD-L1 expressing tumors and only 6.9 months in patients with tumors not expressing PD-L1 [71]. Based on this first cohort of the Keynote-059 trial pembrolizumab gained FDA approval in previously treated PD-L1 positive gastric and GE junction cancer patients. However, optimism was cooled by the results of the Keynote-061 trial randomizing patients between pembrolizumab and an active comparator paclitaxel which did not show any significant benefit [72]. Also, it must be pointed out that paclitaxel as a comparator in the second line of the treatment is a suboptimal standard in this setting with the combination of paclitaxel and ramucirumab being currently the standard of care. Further trials such as Keynote-062 should further clarify the position of the pembrolizumab in the treatment of metastatic gastric cancer. Another checkpoint inhibitor, nivolumab, was investigated in the phase III Attraction-2 trial in heavily pretreated gastric and GE junction adenocarcinoma patients. The patients were randomized regarding treatment with nivolumab or with placebo. The administration of nivolumab extended median overall survival from 4.14 to 5.26 months. This benefit was significant in patients with PD-L1 positive and well as PD-L1 negative tumors [73]. Based on this trial, nivolumab has gained approval in Japan, but not by the FDA or EMA. At this moment, no immunotherapeutic agent is approved for the treatment of esophageal, gastric or GE junction cancer in Europe. Several trials are ongoing at this moment with immune checkpoint inhibitors, both anti-PD1 and anti-PD-L1 antibodies, investigating patients with gastric, GE junction or esophageal cancer not only in advanced/metastatic disease setting, but also in adjuvant, neoadjuvant, or perioperative indications.

## 6. Conclusions

At this moment, PD-L1 expression and MSI-H phenotype, although not ideal, seem to be major prognostic factors as well as predictive biomarkers for immunotherapy efficacy in gastric and esophageal cancer. Continuous research is needed to clarify further predictive factors which could identify the subgroup of patients who will benefit from immunotherapy the most, considering its significant cost, toxicity, and potential induction of the hyper-progression.

## Figures and Tables

**Table 1:** Prognostic significance of PD-L1 expression in gastric and esophageal cancer.

Author	Number of Patients	Type of Cancer	Histology	Disease Stage	Percentage of PD-L1+	Prognosis (PD-L1+)	Prognostic Value	Reference
Böger C et al.	465	gastric cancer	adenocarcinoma	all stages	tumor cells: 30.1% (primary cancer), 60% (liver metastases) immune cells: 88.4% (primary cancer), 73.3% (liver metastases)	better	improved OS and tumor specific survival	[12]
Pereira MA et al.	287	gastric cancer	adenocarcinoma	all stages	8.8%	no impact	no impact	[13]
Tamura T et al.	431	gastric cancer	adenocarcinoma	all stages	29.6%	worse	worse OS for stage II/III	[14]
Zhang L et al.	132	gastric cancer	adenocarcinoma	II/III	50.8%	worse	worse OS	[15]
Eto S et al.	105	gastric cancer	adenocarcinoma	II/III	24.7%	worse	worse OS (statistically non-significant)	[16]
Tsutsumi et al.	90	esophageal cancer	SCC	localized	36.6%	worse	worse OS and relapse free survival	[17]
Kim R et al.	200	esophageal cancer	SCC	localized	33.5%	worse	worse locoregional relapse rate and distant metastasis rate, no change in OS	[18]
Zhang W et al.	344	esophageal cancer	SCC	II/III	tumor cells 14.5%, immune cells 24.7%	better	better OS and DFS only in immune cells PD-L1+	[19]
Zhu Y et al.	133	esophageal cancer	SCC	pT3pN0M0	42.1%	worse	worse DFS and OS	[20]
Jiang Y et al.	428	esophageal cancer	SCC	localized and metastatic	79.7%	worse	worse DFS and OS in radically treated patients	[21]
Jesinghaus M et al.	125	esophageal cancer	SCC	all stages	71% tumor cells, immune cells 87%	better	better OS, DSS and DFS (PD-L1+ tumor cells)	[22]
Chen MF et al.	162	esophageal cancer	SCC	not specified	45.7%	worse	worse treatment response and OS	[23]
Kawazoe A et al.	487	gastric cancer	adenocarcinoma	localized	tumor cells 22.8%, immune cells 61.4	no impact	no impact	[24]
Thompson ED et al.	34	gastric cancer	adenocarcinoma	localized	tumor cells 12%, immune cells 44%	worse	worse PFS and OS	[25]
Yang JH et al.	72	gastric cancer	adenocarcinoma	IV	58.3%	better	better PFS	[26]
Seo AN et al.	116	gastric cancer	adenocarcinoma	localized	tumor cells 49.1%, stromal cells 56.9%	worse	worse DFS, not OS	[27]
Ito S et al.	90	esophageal cancer	SCC	localized	19%	worse	worse OS	[28]
Hsieh CC et al.	150	esophageal cancer	SCC	localized	64%	worse	worse DFS	[29]
Kollmann D et al.	168	esophageal cancer	adenocarcinoma	localized	tumor cells 43.5%, immune cells 69%	better	tumor cells expression - better DFS	[30]
Tanaka K et al.	180	esophageal cancer	SCC	localized	29.4%	worse	worse OS for patients after neoadjuvant chemotherapy	[31]
Li Z et al.	137	gastric cancer	adenocarcinoma	all stages	40.9%	worse	worse OS	[32]
Wang L et al.	550	gastric cancer	adenocarcinoma	all stages	37.3%	no impact	not associated	[33]
Saito R et al.	96	gastric cancer	adenocarcinoma	not specified	tumor cells 34%, stromal cells 45%	worse	worse OS and DSS	[34]
Cho J et al.	78	gastric cancer	adenocarcinoma	all stages	9% tumor cells, 60.3% immune cells	better	better OS (immune cells PD-L1+)	[35]
Ma C et al.	44	gastric cancer	adenocarcinoma	all stages	72%	no impact	not associated	[36]
Koh J et al.	392	gastric cancer	adenocarcinoma	II/III	25%	no impact	not associated	[37]

SCC: squamous cell cancer, OS: overall survival, DFS: disease free survival, PFS: progression free survival, DSS: disease-specific survival.

**Table 2:** Prognostic significance of PD-1/PD-L2 expression in gastric and esophageal cancer.

	Author	Number of Patients	Type of Cancer	Histology	Disease Stage	Percentage of Positivity	Prognosis	Prognostic Value	Reference
PD-1	Chen K et al.	349	esophageal cancer	SCC	localized	33.5%	no impact	no impact	[51]
	Wu Y et al.	340	gastric cancer	adenocarcinoma	all stages	22.6%	better	improved OS	[52]
	Böger C et al.	465	gastric cancer	adenocarcinoma	all stages	primary cancer 53.8%, liver metastases 73.3%	better	improved tumor specific survival	[12]
	Eto S et al.	105	gastric cancer	adenocarcinoma	II/III	26.7%	worse	worse DFS	[16]
	Kollmann D et al.	168	esophageal cancer	adenocarcinoma	localized	tumor cells 77.4%, immune cells 81%	worse	worse OS and DFS	[30]
PD-L2	Seo AN et al.	116	gastric cancer	adenocarcinoma	localized	tumor cells 21.6%, stromal cells 38.8%	no impact	not statistically significant trend towards-improved DFS	[27]
	Hsieh CC et al.	150	esophageal cancer	SCC	localized	42%	no impact	no impact	[29]
	Tanaka K et al.	180	esophageal cancer	SCC	localized	48.3%	worse	worse OS for patients after neoadjuvant chemotherapy	[31]

SCC: squamous cell cancer, OS: overall survival, DFS: disease free survival.

**Table 3:** Prognostic and predictive role of MSI-H phenotype in gastric cancer.

Author	Histology	Stage	Number of MSI-H Patients	MSI-H Frequency	Prognostic Role of MSI-H Phenotype	Prognostic/Predictive Value	Reference
Kim H et al.	adenocarcinoma	all stages	161	9%	better	improved prognosis	[61]
An JY et al.	adenocarcinoma	all stages	170	8.5%	no impact	no benefit from adjuvant chemotherapy in MSI-H patients	[62]
Fang WL et al.	adenocarcinoma	I–III	25	11.7%	better	improved 5-year OS (68% vs. 47.6%, *p* = 0.030), trend towards better DFS at 3 years	[63]
Beghelli S et al.	adenocarcinoma	all stages	83	16%	better	improved survival in stage II patients	[64]
Kim SY et al.	adenocarcinoma	II–III	105	8.2%	better	improved prognosis without chemotherapy	[65]
Smyth EC et al.	adenocarcinoma	localized	20	8.5%	better	worse prognosis when treated with chemotherapy	[59]
Polom K et al.	adenocarcinoma	metastatic	14	8.0%	better	improved OS (15.9 vs. 8 months, *p* = 0.023)	[56]
Giampieri R et al.	adenocarcinoma	metastatic	15	14%	better	improved overall survival	[66]
Corso G et al.	adenocarcinoma	all stages	63	25.2%	better	improved long term survival	[57]
Oki E et al.	adenocarcinoma	all stages	22	9.4%	No prognostic role	No prognostic role	[67]
Falchetti M et al.	adenocarcinoma	localized	27	17%	better	improved survival (*p* = 0.1)	[68]
Marrelli D et al.	adenocarcinoma	all stages	111	23.5%	better	improved 5-year survival (67.6% vs. 35%, *p* < 0.001)	[69]
